# Effects of Miao sour soup on hyperlipidemia in high‐fat diet‐induced obese rats via the AMPK signaling pathway

**DOI:** 10.1002/fsn3.2394

**Published:** 2021-06-23

**Authors:** Hongmei Yang, Jiao Xie, Nanlan Wang, Qianqian Zhou, Yang Lu, Zihan Qu, Huiqun Wang

**Affiliations:** ^1^ School of Public Health, the key Laboratory of Environmental Pollution Monitoring and Disease Control Ministry of Education Guizhou Medical University Guiyang China; ^2^ Guiyang Maternal and Child Healthcare Hospital Guiyang China; ^3^ Laishan District Center for Disease Control and Prevention Yantai China

**Keywords:** AMPK signaling pathway, high‐fat diet, hyperlipidemia, lipogenesis, obesity

## Abstract

Hyperlipidemia is a common characteristic of obese animals. Identifying the factors involved in the regulation of dietary lipid metabolism is the most beneficial way to improve health. Miao sour soup (MSS) is a fermented food made from tomato and red pepper that contains lycopene, capsaicin, and organic acids. We conducted this study to investigate the regulatory functions and mechanisms of MSS on the blood lipid levels of high‐fat diet‐induced obese rats. In our preventive study, rats were fed normal diet (ND1), high‐fat diet (HFD1), HFD + 4 g/kg BW MSS (HFD + LS1), and HFD + 8 g/kg BW MSS (HFD + HS1). We found that MSS significantly reduced the body weight and fat accumulation and improved the blood lipid levels of rats. MSS significantly increased the expression of AMP‐activated protein kinase‐alpha (AMPKα), attenuated the expression of the adipogenic transcription factor sterol regulatory element‐binding protein‐1c (SREBP‐1c), and suppressed the expression of fatty acid synthase (FAS) and acetyl‐CoA carboxylase alpha (ACCα), the critical regulators of hepatic lipid metabolism. Additionally, we also conducted a treatment study, and we grouped rats to receive ND2, HFD2, PC2, HFD + LS2, and HFD + HS2 for another 10 weeks. MSS treatment reduced the body weight, fat deposition, and percentage of lipid droplets and regulated the plasma lipid content. MSS significantly increased the expression of AMPK and alleviated the expression of SREBP‐1c, ACC, and FAS. Taken together, these findings suggest that MSS prevents and treats hyperlipidemia in obese rats by regulating the AMPK signaling pathway.

## Graphical abstract

Obesity and its associated complications are the greatest public health problem worldwide. Presently, many researches are investigating food intervention to prevent and treat obesity and its complications. The fermented food product of Miao, Miao sour soup, can reduce lipid synthesis in the liver by activating the AMPK pathway, thus, ultimately preventing and treating obesity and hyperlipidemia in HFD‐induced rats.

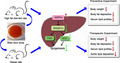

## 1 INTRODUCTION

Obesity is one of the greatest challenges to public health (Aleem et al., 2015). Hyperlipidemia, which mainly manifests as an abnormal increase in serum lipid and/or lipoprotein content (Guo et al., 2018), is a major complication of obesity (Heymsfield & Wadden, 2017). It is usually caused by an unhealthy lifestyle and diet (Li et al., 2019). Recently, due to an increase in the consumption of animal meat, which contains a large amount of energy, saturated fatty acids, and sterols, the risk of obesity and hyperlipidemia has increased (Akiyama et al., 1996). Some drugs, such as atorvastatin and simvastatin, can effectively reduce the plasma lipid levels; however, the use of these drugs is limited due to side effects (Demyen et al., 2013; Molokhia et al., 2008). Previously, it has been reported that fermented foods can improve the levels of triglycerides and cholesterol in obesity (Ali et al., 2018; Deng et al., 2021). Fermented foods have no or few side effects. Among them, Miao sour soup (MSS), a fermented food product that undergoes two fermentation processes, is produced in the southeastern area of Guizhou province. During the first fermentation, tomatoes and red peppers are used as the main ingredients. On completion of the first step, ginger and mountain pepper are added as supplementary materials in the subsequent fermentation step for taste enhancement.

MSS, a traditional food specialty with a history of hundreds of years, has a high nutritional value and health benefits. Previously, we found that MSS contains biologically active ingredients such as lycopene and capsaicin, which amounts to 150 μg/g and 70.6 μg/g of the total content, respectively, and organic acids such as lactic acid and citric acid are produced during fermentation (Yang et al., 2019). Additionally, bioactive food components such as lycopene and capsaicin have lipid‐lowering (Jiang et al., 2015), antioxidative (Gomez‐Sierra et al., 2018), antiatherogenic, and anti‐inflammatory effects (Biddle et al., 2013; Zhang et al., 2019). Furthermore, organic acids produced during the fermentation of MSS have significant antioxidant bioactivities (Lu et al., 2019). Many studies have confirmed that organic acids have a protective role against chronic diseases related to oxidative stress, such as obesity (Lee et al., 2012). Kimchi, a fermented food, has health benefits such as lipid‐lowering and antiatherogenic effects (Park et al., 2014). Studies have shown that the health‐promoting effects of kimchi are closely related to its biologically active compounds and fermentation products (Woo et al., 2017), among which the active compound 3‐(4‐hydroxy‐3,5‐dimethoxyphenyl) propionic acid of kimchi inhibits lipid accumulation by regulating the influx of cholesterol and efflux of associated proteins (Yun et al., 2014). Further, the lactic acid bacteria produced during kimchi fermentation decrease the levels of serum cholesterol (Park et al., 2014). Due to the joint action of these components in MSS, which is a complex fermentation mixture containing both bioactive components and fermentation products, we believe that its beneficial effects on serum lipid profile may improve the lipid levels.

Studies have shown that changes in blood lipid concentrations and lipid synthesis and decomposition are strongly correlated with the liver, which is the central organ associated with lipid metabolism (Ponziani et al., 2015). AMP‐activated protein kinase (AMPK) plays an important role in lipid metabolism in the liver (Chiu et al., 2015). AMPK monitors the energy status of cells, regulates the synthesis and oxidation of fatty acids, and synthesizes and transforms cholesterol (Gomez‐Lechon et al., 2007). The elevation of AMP/ATP ratio activates AMPK through phosphorylation at threonine 172 (Thr172) in the α‐subunit (Foretz et al., 2010). Activated AMPK then inhibits various lipid metabolism target genes, including sterol regulatory element‐binding protein‐1c (SREBP‐1c), fatty acid synthase (FAS), and acetyl‐CoA carboxylase alpha (ACCα) (Lu et al., 2018; Snel et al., 2012). Thus, we assumed that the AMPK signaling pathway might also play an important role in modulating the lipogenesis‐related gene/protein expression in liver tissues after MSS supplementation. Accordingly, we conducted this study to explore the preventive and therapeutic effects of MSS on the lipid levels of diet‐induced obese rats from the aspects of body weight, fat accumulation, and serum lipid levels, to investigate the gene and protein expression of lipogenesis‐related factors in the liver that are regulated by AMPK, and to decipher the preliminary molecular mechanism of blood lipid regulation.

## 2 MATERIALS AND METHODS

### 2.1 Materials

MSS was purchased from Mingyang Food Co., Ltd. under the commercial brand name YuMeng. Zhibituo, a drug used in the clinical treatment of hyperlipidemia, was obtained from the Chengdu Diao Jiuhong Pharmaceutical Co., Ltd. Antibodies against AMPKα, ACC, and FAS were purchased from Cell Signaling Technology, and SREBP‐1c and β‐actin antibodies were purchased from Affinity Biosciences.

### 2.2 Preparation of MSS and Zhibituo solution

MSS stock solution was diluted with distilled water to a concentration of 0.8 g/ml, and it was heated to boil. MSS mixture was then filtered using six layers of gauze to obtain the filtrate. Zhibituo tablets were weighed and pulverized. Subsequently, 0.5 g of the powder was accurately weighed and transferred into a 50 ml beaker. The drugs were dissolved in distilled water, and they were filtered with a 0.22 µm filter. The supernatant was collected, and distilled water was added to make up the volume to 50 ml.

### 2.3 Animals and treatment

All animal experiments were conducted in accordance with the Affairs Concerning Experimental Animals, and they were approved by the Guizhou Medical University Animal Ethics Committee. Male Sprague Dawley (*SD*) rats (weight: 180 ± 20 g) were obtained from the Guizhou Medical University Animal Center, and they were raised under normal conditions without specific pathogens at 20–25℃ under 50%‐70% humidity and a 12‐hr light/dark cycle. Prior to the experiments, all rats were adaptively fed for a week with five or six rats per cage. For preventive and therapeutic studies, the rats were transferred to new cages such that two rats were present per cage. First, a preventive study was conducted. Thirty‐two *SD* rats were divided into the following four groups (*n* = 8 per group): normal diet (ND1), high‐fat diet (HFD1), HFD + 4 g/kg BW MSS (HFD + LS1), and HFD + 8 g/kg BW MSS (HFD + HS1). Standard feed was provided by the Guizhou Medical University Animal Center, and HFD comprised 78.8% standard feed, 1% cholesterol, 10% lard, 10% egg yolk powder, and 0.2% bile salt from pigs. The equivalent animal dosage was determined using the equivalent human dose. According to the dietary intake interviews, the usual dosage of MSS is approximately 10 g/per dose for adult humans. The calculated animal dose was 8 g/kg in rats. The MSS dose for this study was 4 or 8 g/kg BW per day. Rats in the HFD + LS1 and HFD + HS 1 groups were gavaged with 4 g/kg BW and 8 g/kg BW MSS, respectively, and those in ND1 and HFD1 groups were gavaged with the same volume of distilled water. MSS and distilled water were administered at 1 ml/100 g (BW) per day. The body weights of rats were measured every week. The preventive study lasted for 10 weeks. At the end of the 10th week, rats were anesthetized via an intraperitoneal injection of pentobarbital sodium (0.5 ml/100 g BW) to collect the blood sample and liver tissue. In the second part, the therapeutic study was performed. Another 40 *SD* rats were randomized into two groups: normal diet (ND2) (*n* = 8) and obesity model (OB) (*n* = 32) groups. The ND2 group was fed normal diet, and the OB group was fed HFD for approximately 12 weeks when the body weight and serum lipid levels of the two groups were significantly different. Obesity models were successfully established. The 32 obese rats were randomized into the following groups: HFD group (HFD2), HFD + 75 mg/kg BW Zhibituo solution (positive control, PC2), HFD + 4 g/kg BW MSS (HFD + LS2), and HFD + 8 g/kg BW MSS (HFD + HS2); at this time, the ND2 rats were still retained. The ND2 and HFD2 groups were gavaged with distilled water, PC2 group was gavaged with Zhibituo solution, and HFD + LS2 and HFD + HS2 groups were gavaged with different dosages of MSS according to the dose design. MSS and distilled water were administered at 1 ml/100 g (BW) per day. The therapeutic experiment lasted for ten weeks. The daily food consumption of rats was noted, and the body weights of rats were measured every week. At the end of the 10th week, rats were anesthetized via an intraperitoneal injection of pentobarbital sodium (0.5 ml/100 g BW), and blood samples, liver tissues, and aorta tissues were obtained.

### 2.4 The index of tissue measurement

The liver index, Lee's index, and adiposity index were computed using the following equations: liver index = (liver weight/body weight) × 100, Lee's index =body weight (g)^1/3^ / nose‐to‐anus length (cm) × 1,000 (Novelli et al., 2007), and adiposity index = intra‐abdominal fat (g) / body weight (g) × 100 (Hao et al., 2015).

### 2.5 Biochemical analysis

Blood was collected into blood collection tubes, centrifuged at 1,800 × g and 4℃ for 15 min using a low‐speed tabletop centrifuge (Shanghai Anting Scientific Instrument Factory, TDL‐5000bR, Shanghai, China) to obtain serum, and stored at −20℃. Serum triacylglycerol (TG), total cholesterol (TC), low‐density lipoprotein cholesterol (LDL‐C), and high‐density lipoprotein cholesterol (HDL‐C) levels were measured using an automatic biochemical analyzer (Beckman Coulter, Lx‐20, Brea).

### 2.6 Histological analysis

The aorta was removed and fixed in 4% paraformaldehyde, and the slides were washed with 60% isopropanol once. Following this, the sections were stained using a freshly prepared Oil Red O working solution for 15 min, and they were counterstained with hematoxylin. Image‐Pro Plus software (version 6.0) was used for image analysis. The results are presented as the ratio of total lipid droplet area to total vessel area.

### 2.7 Quantitative reverse transcription polymerase chain reaction (qRT‐PCR) analysis

Total RNA from rat liver tissues was extracted using TRIzol reagent. After further purification, the purity and concentration of RNA were determined using spectrophotometry, and RNA integrity was checked via agarose gel electrophoresis. Following this, cDNA was prepared using the PrimeScript RT reagent Kit with gDNA Eraser. The primer sequences used are listed in Table 1. qRT‐PCR was performed using a SYBR Premix Ex Taq^TM^ Kit on a CFX96 Real‐Time PCR System (Bio‐Rad, Hercules, CA, USA). β‐actin was used as the housekeeping gene for normalization of targeted gene quantities. Relative quantification of gene expression was determined using the *ΔΔCT* method (where CT is the threshold cycle). The relative expression levels of target genes were calculated using the 2^−ΔΔCt^ formula.

**TABLE 1:** Primers for real‐time PCR analysis

Primer	Forward primer	Reverse primer
AMPKα	CCCGACACACCCTAGATGAA	TGCTCTACACACTTCTGCCA
SREBP−1c	CCCACCTCAAACCTGGATCT	TCTCAGCCTGTAGTCCCTCT
ACC	ACATCGGTCCTGTGTCAGTT	TCCATCACCACAGCCTTCAT
FAS	ACCTGGTGACCCTGAATCTG	CTTTCCGGGATCTTGTGCTG
β‐actin	TGTCACCAACTGGGACGATA	GGGGTGTTGAAGGTCTCAAA

### 2.8 Western blot analysis

After collecting the liver samples, total protein was extracted with radio immune‐precipitation assay (RIPA) lysate containing phenylmethanesulfonyl fluoride (PMSF) (PMSF:RIPA = 1:100). Following centrifugation of tissue homogenates at 13,680 × g and 4℃ for 15 min, the supernatant was collected for assessment of protein concentration using the BCA protein concentration assay kit. The samples were subjected to sodium dodecyl sulfate polyacrylamide gel electrophoresis, and subsequently, they were transferred to polyvinylidene difluoride membranes. Membranes were blocked with 5% skim milk at room temperature for 2 hr. Next, the membranes were incubated with specific primary antibodies at 4℃ overnight. After incubation, the membranes were washed thrice with Tris‐buffered saline with Tween‐20 (TBST), and they were incubated with the secondary antibody for 2 hr. After rinsing with TBST, bound antibodies were visualized using an ECL kit, and they were quantified using the ImageJ software.

### 2.9 Statistical analysis

Statistical analysis was conducted using SPSS (version 23.0; SPSS Inc., Chicago, IL, USA). Student's *t* test was used for comparisons between two groups. For comparison of data between three or more groups, data were analyzed using analysis of variance (ANOVA). When ANOVA showed a significant difference, the differences were further evaluated using the least significant difference. Statistical significance was set at *p* < .05.

## 3 RESULTS

### 3.1 The preventive effect of MSS on hyperlipidemia in HFD‐induced obese rats

#### 3.1.1 MSS reduces the body weight and body fat deposition and improves the serum lipid levels of HFD‐fed rats

**FIGURE 1:**
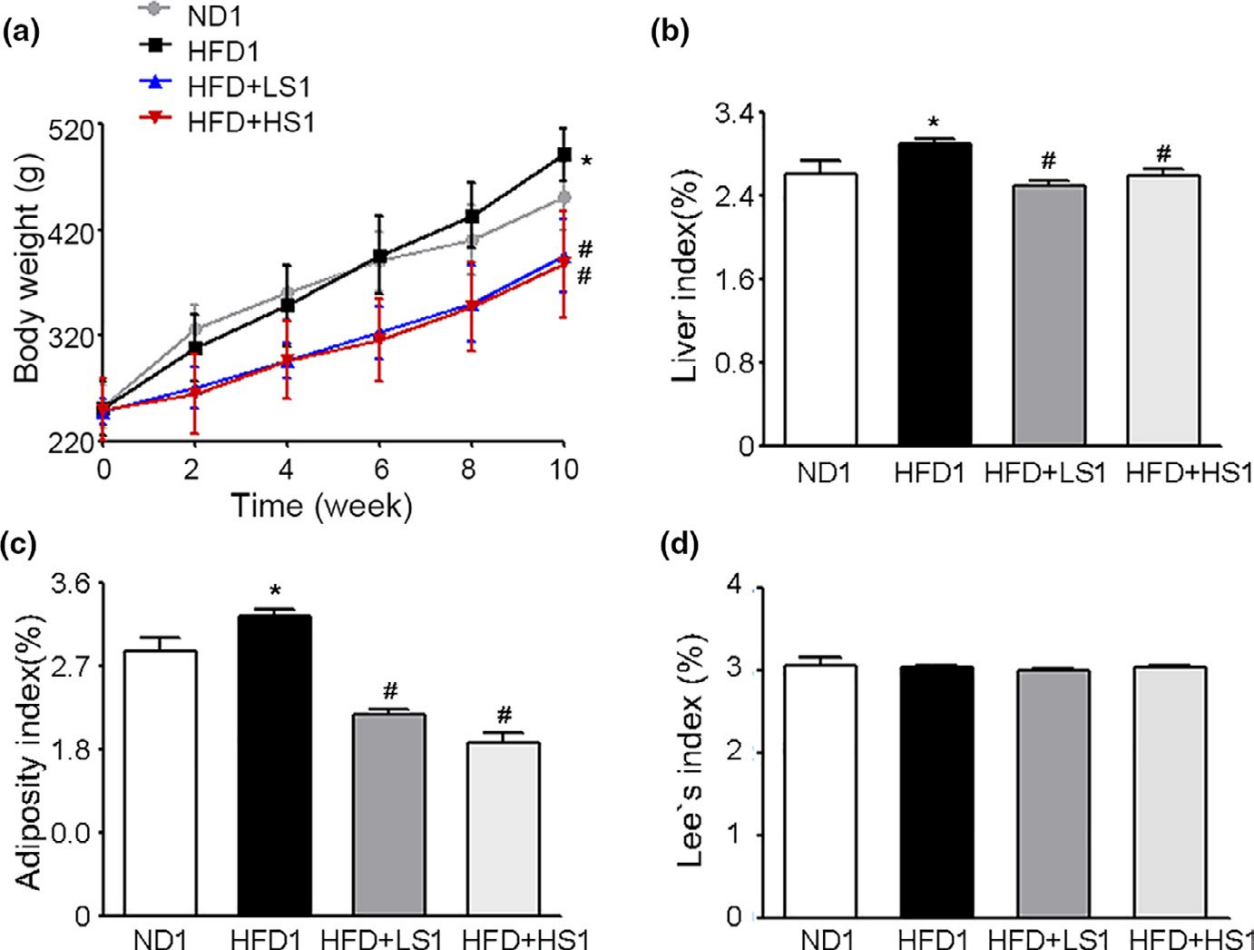
Effect of Miao sour soup (MSS) on the Body Weight and Fat Deposition of high‐fat diet (HFD)‐fed Rats. Rats were fed a normal diet (ND1), HFD (HFD1), HFD supplemented with 4 g/kg BW MSS (HFD + LS1), or HFD supplemented with 8 g/kg BW MSS (HFD + HS1) for 12 weeks. (a) Body weights of rats during the first 10 weeks. (b) Liver index, (c) Adiposity index, and (d) Lee's index are presented as the mean ± *SD* (*n* = 8). (∗) *p* < .05, versus ND1 group, (#) *p* < .05, versus the HFD1 group

The effect of MSS on the body weight and body fat accumulation of HFD‐fed rats was evaluated. As shown in Figure 1, at the end of the 10th week, compared with the weight of ND1 group (451.61 ± 32.09), the weight of HFD1 group (491.56 ± 24.69) was higher, and the weights of HFD + LS1 (395.15 ± 34.62) and HFD + HS1 (391.17 ± 47.16) groups were lower than that of HFD1 group (*p* < .05). HFD + LS1 and HFD + HS1 groups showed less weight gain. Furthermore, the liver index and adiposity index of HFD1 group were significantly higher than those of ND1 group while the liver index and adiposity index of HFD + LS1 and HFD + HS1 groups were significantly lower than those of HFD1 group (*p* < .05) (Figure 1b,c). The Lee index did not show a significant difference among the four groups (*p* > .05) (Figure 1d). These results showed that MSS administration decreased the body weight gain and body fat accumulation in HFD‐fed rats.

**FIGURE 2:**
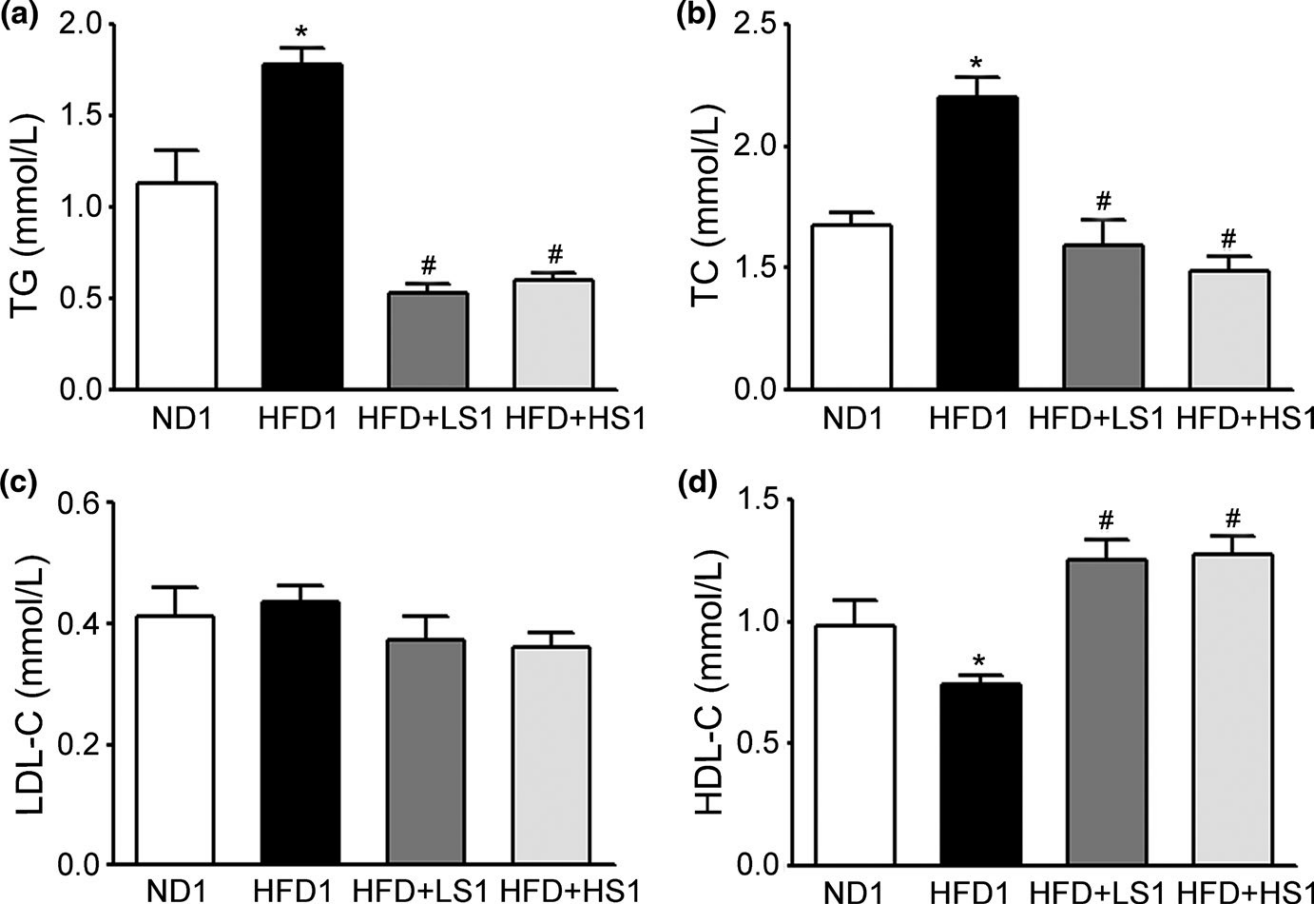
Effect of MSS on the Serum Lipid Levels of HFD‐Fed Rats. (a) Triglyceride (TG), (b) total cholesterol (TC), (c) low‐density lipoprotein cholesterol (LDL‐C), and (d) high‐density lipoprotein cholesterol (HDL‐C) levels. Results are presented as the mean ± *SD* (*n* = 8). (∗) *p* < .05, versus ND1 group, (#) *p* < .05, versus HFD1 group

Changes in the serum lipid levels of obese rats were examined to evaluate the effect of MSS on lipid metabolism. As shown in Figure 2, serum TG and TC levels were significantly higher while the HDL‐C concentration was significantly lower in HFD1 group than in ND1 group (*p* < .05). HFD + LS1 and HFD + HS1 groups exhibited decreased serum TG and TC levels and an elevated HDL‐C concentration (*p* < .05). The serum LDL‐C levels of all tested groups did not show a significant difference (*p* > .05). The results showed that MSS could effectively alleviate lipid disorders in HFD‐fed rats.

#### 3.1.2 MSS inhibits adipogenesis in HFD‐fed rats via the AMPK signaling pathway

**FIGURE 3:**
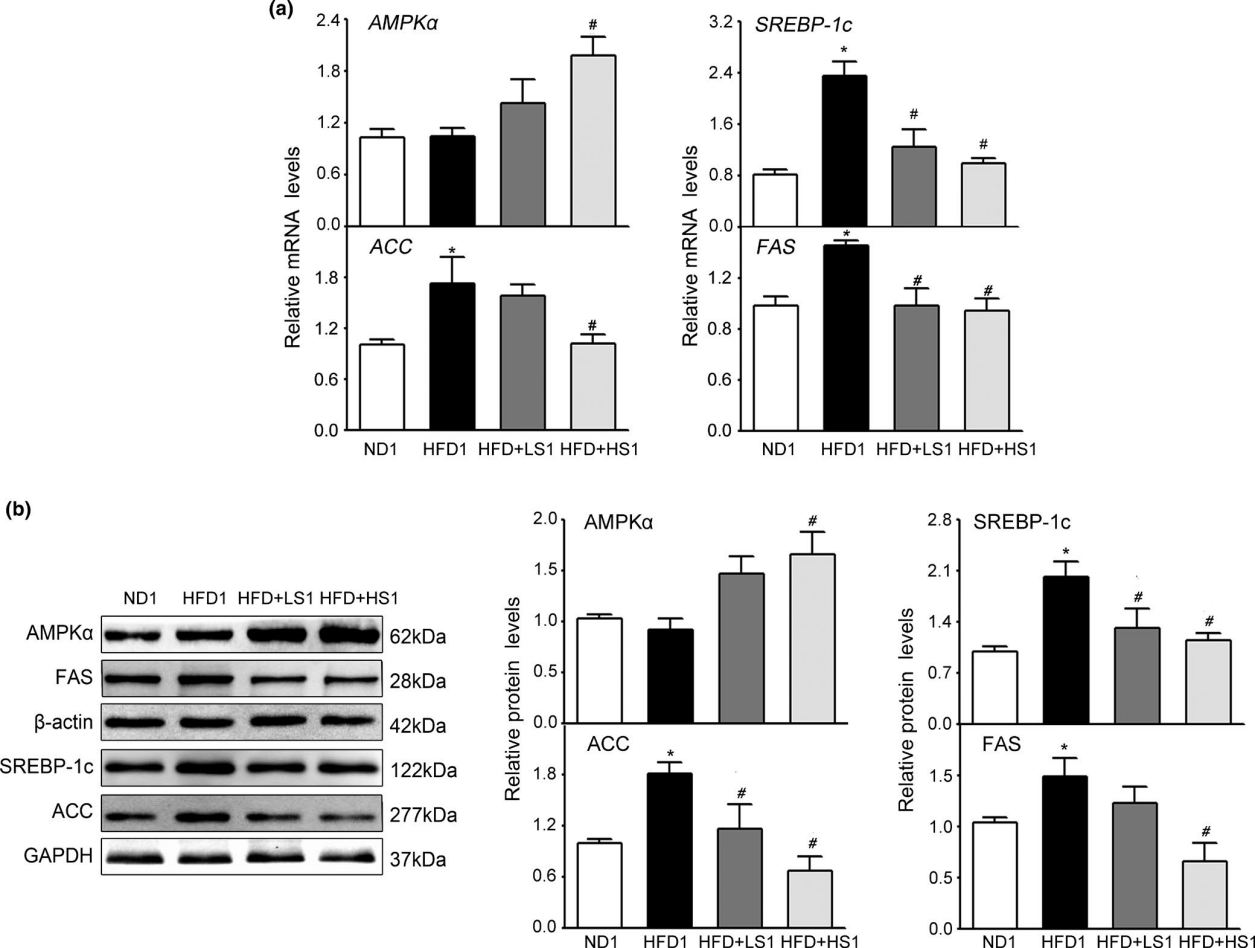
Effects of MSS on Lipogenesis‐Associated Genes and Proteins in the Liver of HFD‐fed Rats. Gene expression in the liver was analyzed via quantitative real‐time PCR (qRT‐PCR), and protein extracts from the liver were analyzed via Western blotting (WB). (a) qRT‐PCR analyses of live lipid‐related molecules such as AMP‐activated protein kinase (AMPK), sterol regulatory element‐binding protein‐1c (SREBP‐1c), fatty acid synthase (FAS), and acetyl‐CoA carboxylase alpha (ACCα). (b) Western blot analysis of lipid‐related molecules (AMPKα, SREBP‐1c, ACCα, and FAS). Results are presented as the mean ± *SD* (*n* = 8). (∗) *p* < .05, versus ND1 group, (#) *p* < .05, versus the HFD1 group

We examined the impact of MSS on the expression of rat adipogenesis‐related genes and proteins regulated by AMPK. As shown in Figure 3a, no significant effect was observed on *AMPKα* gene expression in HFD1 group than in ND1 group (*p* > .05). Compared with the ND1 group, the HFD1 group exhibited a significantly increased expression of *SREBP‐1c*, *ACC*, and *FAS* genes while HFD + LS1 and HFD + HS1 groups significantly suppressed the expression of these genes and increased the expression of *AMPKα* gene as compared to the HFD1 group (*p* < .05). Furthermore, at the protein expression level, HFD induced the expression of SREBP‐1c, ACC, and FAS proteins. MSS supplementation upregulated the protein expression levels of AMPKα and decreased the expression of SREBP‐1c, ACC, and FAS proteins (*p* < .05) (Figure 3b). The results showed that MSS could activate AMPK expression, which inhibited the expression of SREBP‐1c, ACC, and FAS, thereby suppressing hepatic lipogenesis in rats.

### 3.2 The therapeutic effect of MSS on hyperlipidemia in obese rats

#### 3.2.1 HFD‐induced obesity

**FIGURE 4:**
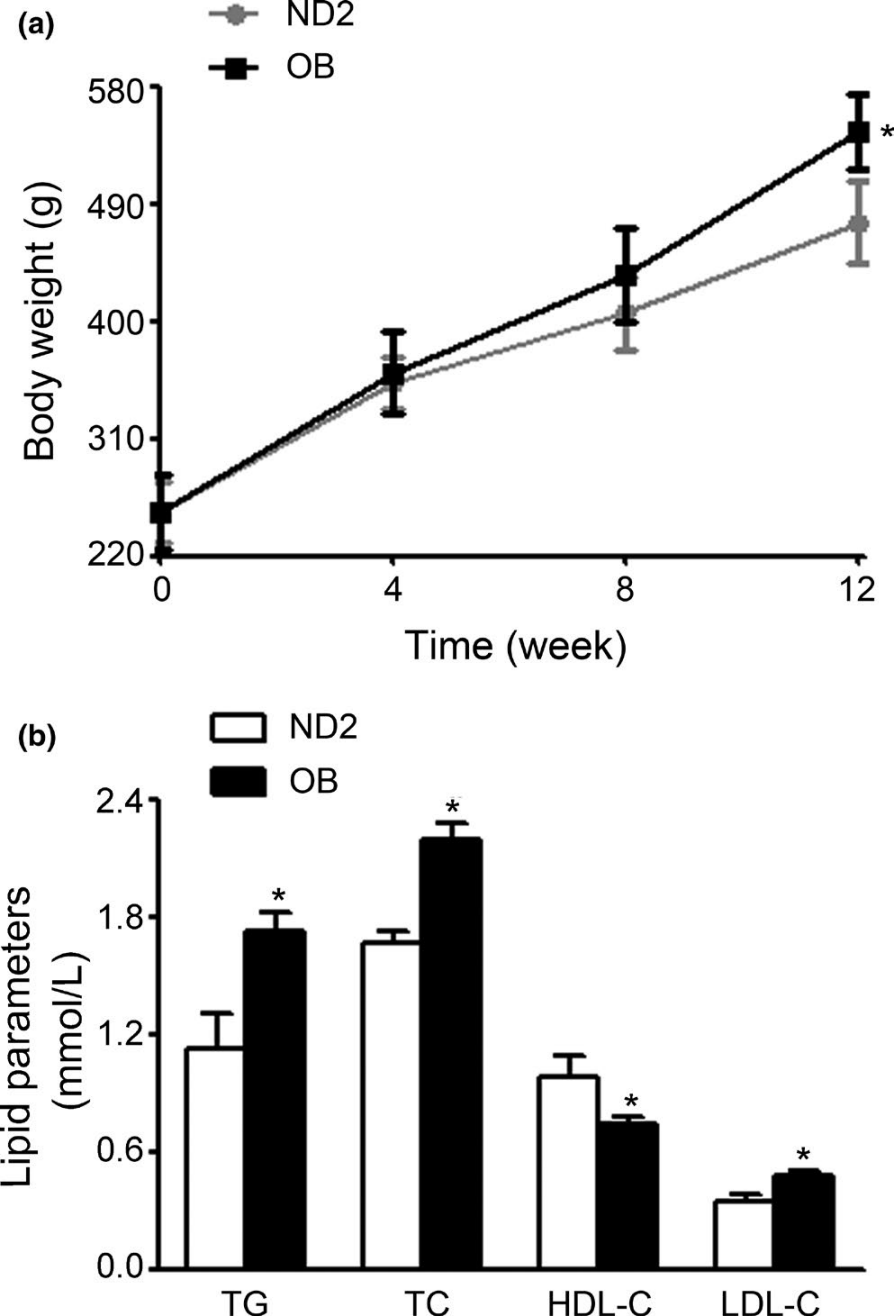
Establishment of obese rats model. Rats were fed a normal diet (ND2) and HFD (OB) for 12 weeks. (a) Body weights. (b) TG, TC, LDL‐C, and HDL‐C levels

The rats were fed HFD, and the body weight and serum lipid profiles were analyzed at 12 weeks. As showed in Figure 4a significant increase was observed in the body weights of OB group (538.65 ± 19.84) than that of ND2 group (476.08 ± 31.56) (*p* < .05). Serum TG, TC, and LDL‐C levels increased whereas HDL‐C levels significantly decreased in OB group than in ND2 group (Figure 4b). These results indicated the successful establishment of HFD‐induced obesity and hyperlipidemia.

#### 3.2.2 MSS reduces the body weight and fat accumulation and improves the lipid profiles of obese rats

**FIGURE 5:**
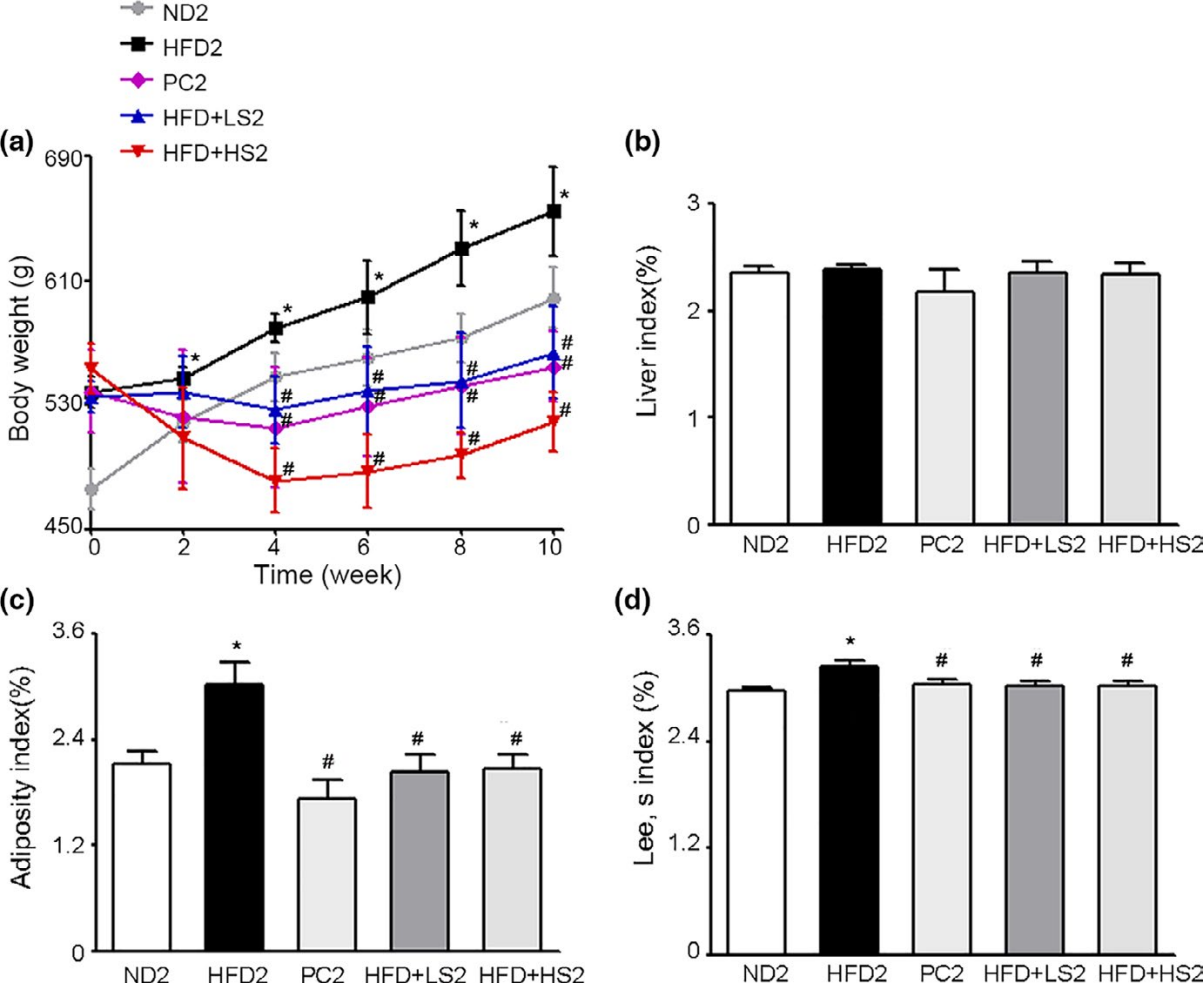
Effects of MSS on the body weight and body fat accumulation of obese rats. After 12 weeks of HFD induction, the hyperlipidemic rats were fed a normal diet (ND2), high‐fat diet (HFD2), HFD + 75 mg/kg BW Zhibituo solution (positive control, PC2), HFD supplemented with 4 g/kg BW MSS (HFD + LS2), or HFD supplemented with 8 g/kg BW MSS (HFD + HS2) for 10 weeks. (a) Body weights, (b) Liver index, (c) Adiposity index, and (d) Lee's index are presented as the mean ± *SD* (*n* = 8). (∗) *p* < .05, versus ND2 group, (#) *p* < .05, versus HFD2 group

The effects of MSS on the body weight and body fat accumulation of obese rats were evaluated. First, we observed no visible differences in the food intake of each group (*p* > .05). As shown in Figure 5a, during the entire study period, the body weights of HFD2 rats were significantly higher than those of ND2 rats. At the forth week, rats supplemented with Zhibituo solution (PC) or MSS (LS and HS) showed a significant decrease in body weight as compared to HFD2 rats, and the increased body weights of HFD2 rats decreased during the following 6 weeks (*p* < .05). Body weights of PC2, HFD + LS2, and HFD + HS2 groups were significantly lower than those of HFD2 group at the end of the 10‐week intervention (*p* < .05). Additionally, no significant difference was observed in the liver indices of all groups (Figure 5b) while supplementation with Zhibituo solution (PC) or MSS (LS and HS) significantly reduced the adiposity index and Lee's index as compared to those of HFD2 group (*p* < .05) (Figure 5c,d). Our results showed that MSS has potential anti‐obesity effects that reduce the body weight and body fat accumulation of rats without affecting the food intake.

**FIGURE 6:**
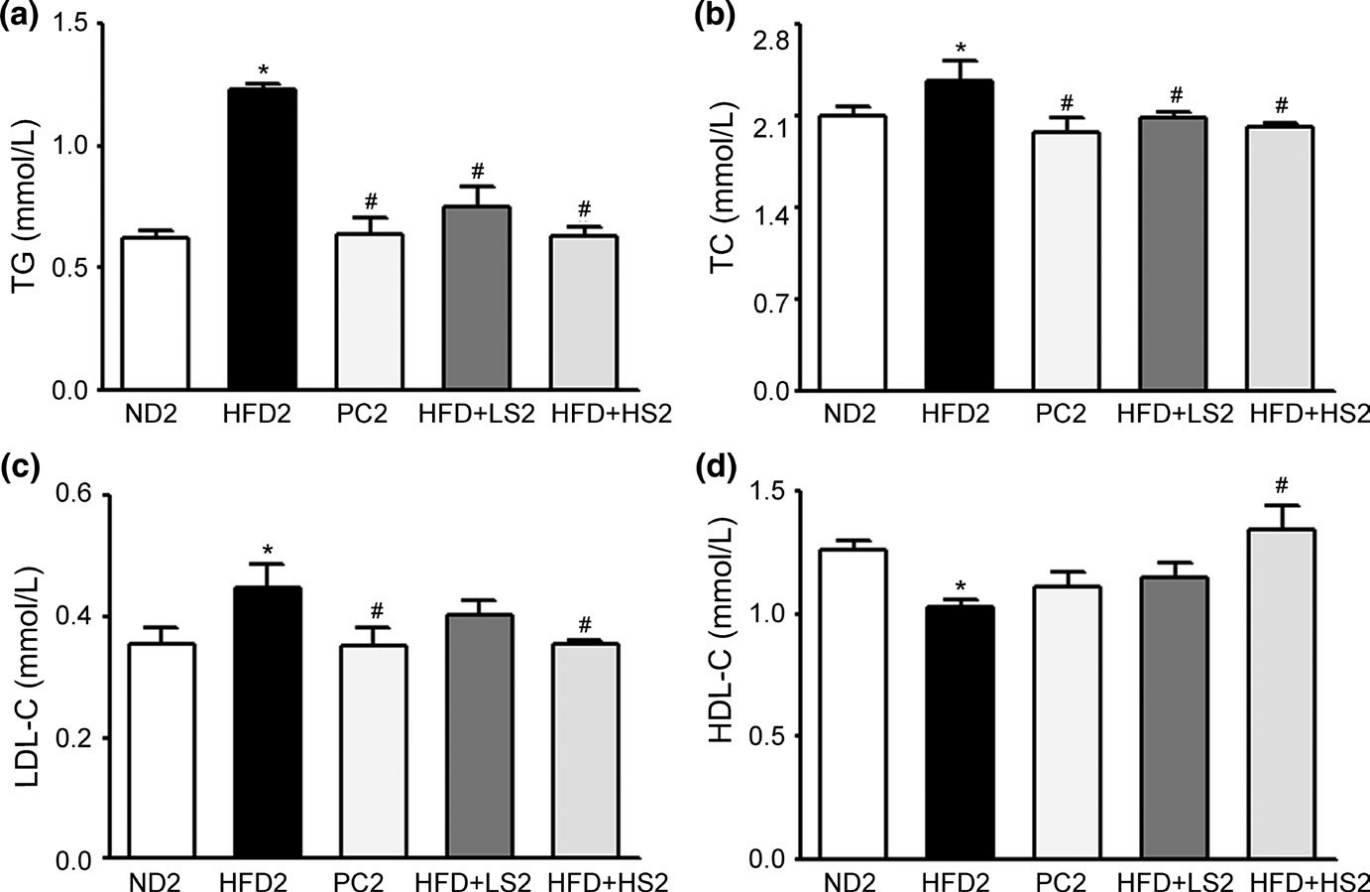
Effects of MSS on the serum lipid levels of obese rats. (a) TG, (b) TC, (c) LDL‐C, and (d) HDL‐C levels. Results are expressed as the mean ± *SD* for each group (*n* = 8). (∗) *p* < .05, versus ND2 group, (#) *p* < .05, versus HFD2 group

The effect of MSS on the serum lipid levels of obese rats was evaluated. As shown in Figure 6, the serum levels of TG, TC, and LDL‐C significantly increased and serum HDL‐C levels decreased in HFD‐induced obese rats than in ND2 rats. PC2, HFD + LS2, and HFD + HS2 groups showed decreased TG and TC levels, and PC2 and HFD + HS2 groups showed decreased LDL‐C concentrations whereas HFD + HS2 group showed an increased HDL‐C concentration (*p* < .05). Furthermore, TG and TC levels in HFD + LS2 and HFD + HS2 groups were similar to those in PC2 group (*p* > .05). These results suggest that MSS has the same therapeutic effects as hypolipidemic drugs in the treatment of hyperlipidemic rats.

**FIGURE 7:**
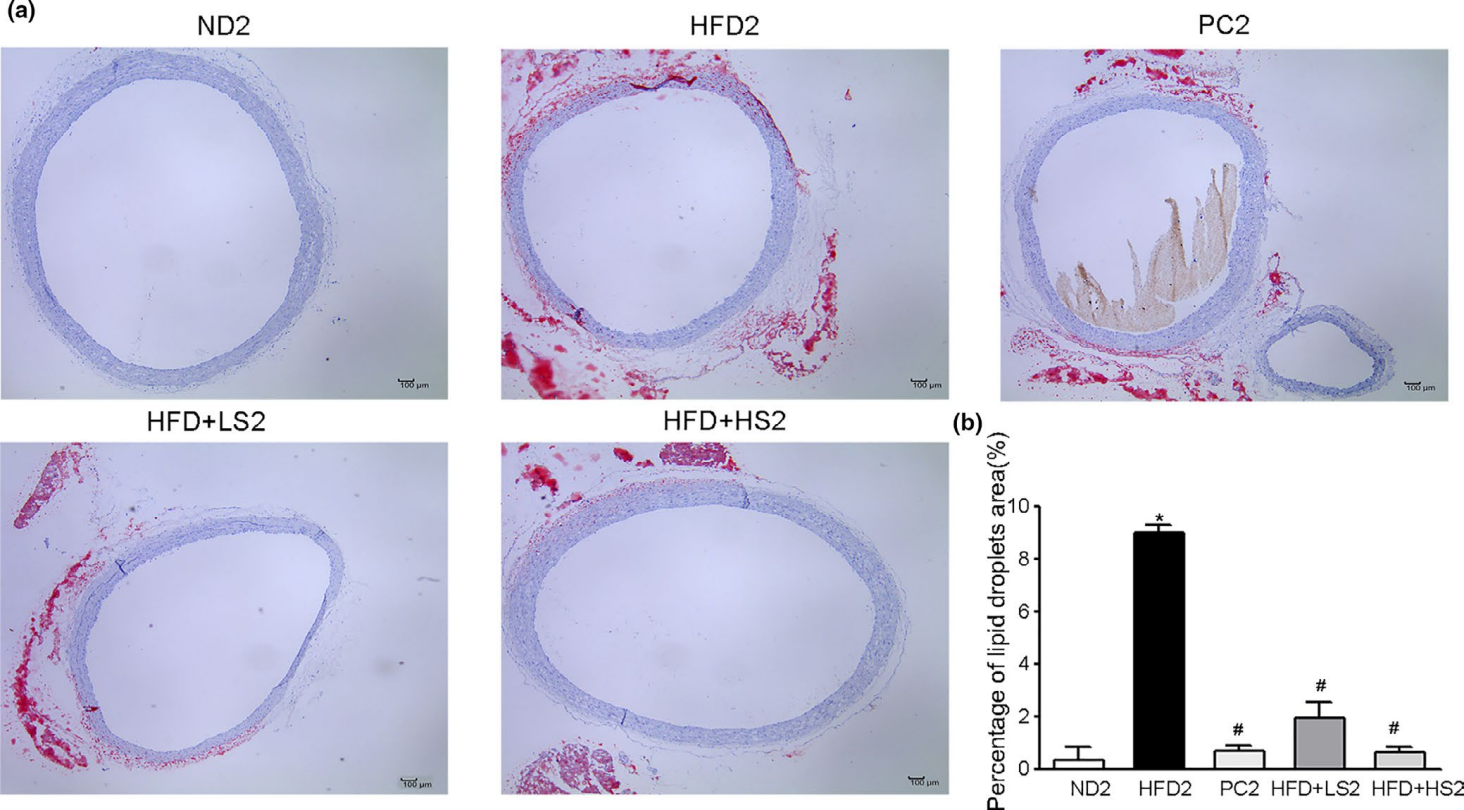
Effect of MSS on aortic lipid deposition in obese rats. (a) Representative photographs of the thoracic aorta section stained with oil red O. (b) Percentage of lipid droplets. (∗) *p* < .05, versus ND2 group, (#) *p* < .05, versus HFD2 group

To further confirm the potential therapeutic effects of MSS, aortic sections were subjected to Oil Red O staining to assess the effect of MSS on aortic lipid deposition. The HFD2 group showed prominent lipid infiltration, and the percentage of lipid droplets in HFD2 group was higher than that in ND2 group. PC2, HFD + LS2, and HFD + HS2 treatment significantly reduced HFD‐induced lipid infiltration and decreased the percentage of lipid droplets (*p* < .05) (Figure 7a,b). The results showed that MSS treatment substantially attenuated lipid deposition in the aorta.

#### 3.2.3 MSS inhibits adipogenesis through the AMPK signaling pathway in obese rats

**FIGURE 8:**
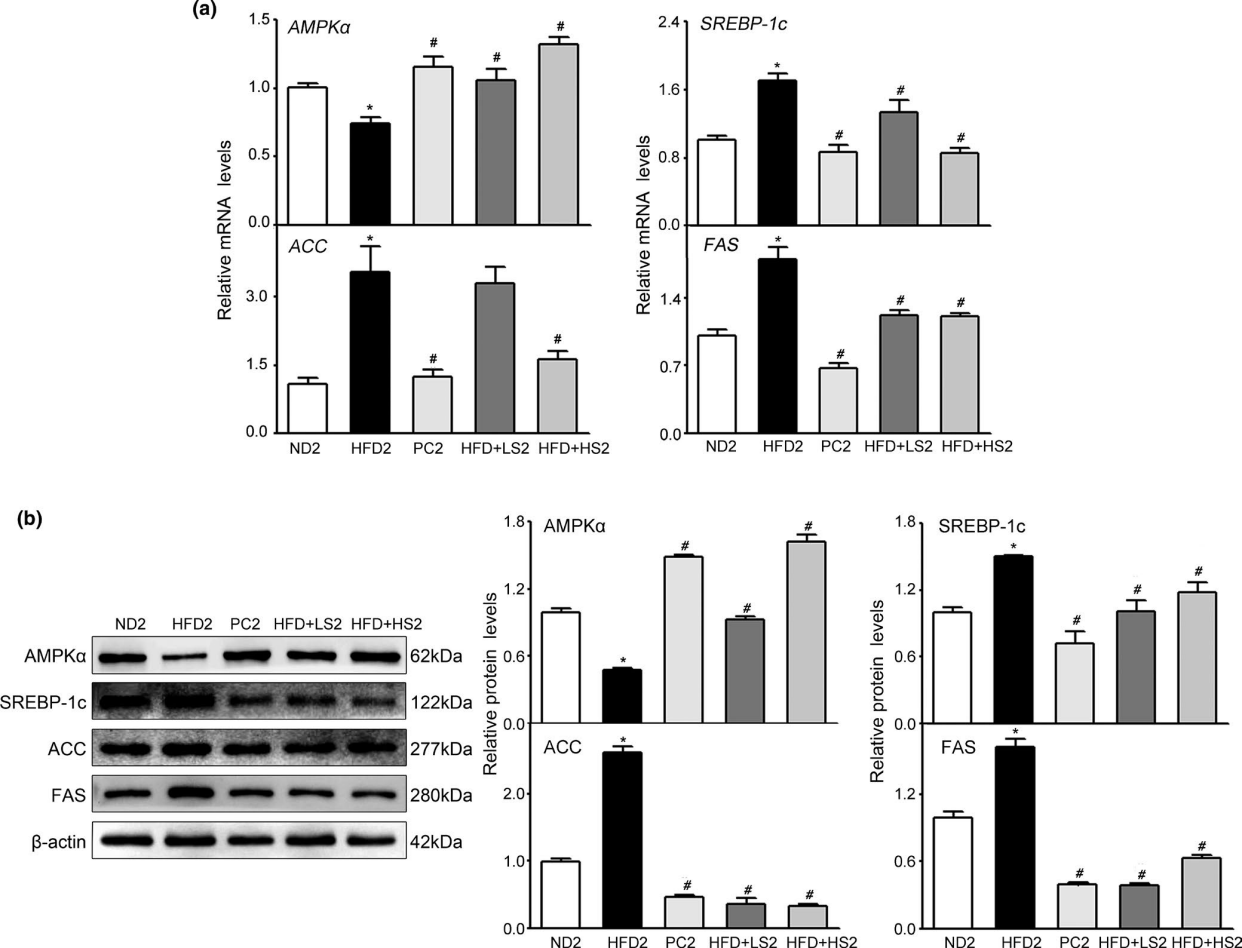
Effects of MSS on lipogenesis‐related factors in the livers of obese rats. Gene expression in the liver was analyzed via qRT‐PCR, and protein extracts from the liver were measured via WB. (a) qRT‐PCR analyses of live lipid‐related molecules (AMPKα, SREBP‐1c, ACC, and FAS). (b) Western blot analysis of lipid‐related molecules (AMPKα, SREBP‐1c, ACC, and FAS). Results are presented as the mean ± *SD* (*n* = 8). (∗) *p* < .05, versus ND2 group, (#) *p* < .05, versus HFD2 group

As shown in Figure 8a, gene expression of the cellular energy sensor *AMPKα* was significantly lower while the expression of other target genes of the AMPK signaling pathway, *SREBP‐1c, ACC,* and *FAS* was significantly higher in HFD2 group than in ND2 group. Supplementation with Zhibituo solution (PC) or MSS (LS and HS) significantly increased the expression levels of *AMPKα* while supplementation with Zhibituo solution (PC) or MSS (HS) significantly decreased the expression of *SREBP‐1c, ACC,* and *FAS* (*p* < .05). Moreover, at the protein level, compared with HFD2 group, PC2, HFD + LS2, and HFD + HS2 groups showed significantly increased expression levels of AMPKα protein and decreased expression levels of SREBP‐1c, ACC, and FAS proteins (*p* < .05) (Figure 8b).

## 4 DISCUSSION

This study is the first to reveal the preventive effect of MSS on the development of HFD‐induced obesity and hyperlipidemia. We also established an obesity model to investigate the therapeutic effect of MSS on hyperlipidemia in obese rats. When discrepancies occurred, changes in the levels of genes and proteins associated with the AMPK/SREBP pathway after MSS administration were investigated to elucidate the anti‐obesity and hypolipidemic mechanisms of MSS and to provide a theoretical basis for the prevention and treatment of obesity with MSS.

As a typical fermented food in Guizhou Province, MSS is thought to exert beneficial effects on lipid metabolism. However, few reports have focused on the preventive and therapeutic effects of MSS on obesity and its potential mechanisms. Therefore, we first examined the preventive effect of MSS on the development of obesity and hyperlipidemia in HFD‐fed rats. We found that the body weight, liver index, adiposity index, and serum TC and TG levels significantly increased while HDL‐C levels significantly decreased in HFD1 group. HFD can lead to liver enlargement and lipid accumulation while the increase in liver index and adiposity index indicates the presence of obesity to a certain extent (Cho et al., 2017). Obesity is commonly accompanied by disorders in lipid metabolism, and its most common presentation is abnormally elevated serum lipid levels (Du et al., 2020). Thus, serum TC, TG, LDL‐C, and HDL‐C levels are key indicators for evaluating lipid metabolism (Dechesne et al., 2016). The increase in serum TG, TC, and LDL‐C levels increases the incidence of cardiovascular diseases (Sarwar et al., 2007). Our results proved that obesity and hyperlipidemia were induced in HFD‐fed rats, which was consistent with previous reports (Liu et al., 2019). After the intervention, MSS decreased the body weight gain, liver index, and adiposity index in HFD‐fed rats. Additionally, the increase in TG and TC levels and decrease in HDL‐C levels were inhibited in MSS‐fed rats, suggesting that MSS effectively decreased the body weight gain and regulated blood lipid metabolism in HFD‐fed rats. Capsaicin has been reported to inhibit lipid accumulation in fat cells and experimental animals as well as reduce white fat pad mass; thus, capsaicin has anti‐obesity effects (Hwang et al., 2005). Son et al. reported that the fermented food gochujang, which contains many bioactive compounds such as capsaicin and soybean fermentation products, decreases the body weight gain and weights of the liver and white fat pads in HFD‐fed rats (Son et al., 2020). Andrea et al. showed that capsaicin improves blood lipid levels and reduces hepatic fat accumulation in Western diet‐fed rats (Andrea et al., 2018). Additionally, lycopene has been found to reduce serum cholesterol levels (Purwantoyo et al., 2018). Therefore, the reduction in body weight gain and improvement in the blood lipid profile of rats induced by MSS are likely due to the biologically active compounds, such as lycopene, capsaicin, and fermentation products, present in MSS.

In the preventive study, rats fed HFD and MSS showed a significant decrease in the body weight relative to the HFD1 group. Therefore, we could not rule out the possibility that the decreased body weight gain indirectly induced the attenuation of serum lipids. As MSS has a certain degree of sourness and spicy flavor, a higher gavage dose may lead to gastrointestinal irritation, resulting in a decreased food intake and weight loss. Consequently, to ensure the therapeutic effect of MSS on hyperlipidemia in obese rats, diet‐induced obese rats were fed for 12 weeks and then fed with HFD and different concentrations of MSS for 10 weeks. The food consumption of each group of rats was measured daily, and the daily energy intake per rat was calculated based on the total energy intake. However, no corresponding changes were observed in the daily food intake of each group. Our results suggested that MSS could reduce the body weight, adiposity index, and Lee's index in obese rats without affecting the food intake. Considering the similar food intake among the groups, MSS administration lowered the body weight and body fat accumulation in HFD + LS2 and HFD + HS2 groups than in HFD2 group. Obesity develops when the energy intake exceeds the energy expenditure (Abreu‐Vieira et al., 2015). In this study, no visible differences were observed in the food intake of each group. These results suggest that MSS reduced the body weight in obese rats possibly by inhibiting the digestion/absorption of food, suppressing the lipid production, and increasing the energy expenditure (Choo, 2000). The results showed that MSS could reduce the serum TC, TG, and LDL‐C levels whereas it could increase HDL‐C levels in obese rats, and the hypolipidemic efficacy of MSS was comparable to that of hypolipidemic drugs. Furthermore, MSS treatment substantially attenuated lipid deposition in the aorta. Clinical trials have shown that serum TC, TG, HDL‐C, and LDL‐C levels are related to the development of atherosclerosis (Hou et al., 2018). Nevertheless, the key characterization of atherosclerosis is the accumulation of lipid deposits within the intimal layer of arteries (Emini et al., 2017). Thus, plasma TG and TC levels are closely related to aortic lipid deposition, and plasma lipid levels may indicate deposition in aortic tissues (West et al., 1983). Therefore, the present results showed that MSS reduced lipid deposition in the aorta, which further proved the efficacy of MSS in regulating lipid metabolism. Obesity and its associated morbidity, such as hyperlipidemia, pose a major hazard to public health (Aleem et al., 2015). However, the currently available antihyperlipidemic drugs have serious adverse reactions. For example, the hypolipidemic drug simvastatin can cause severe adverse events, such as rhabdomyolysis (Omar & Wilson, 2002). Fermented foods are being investigated and used to maintain our health due to the adverse effects of synthetic chemicals and drugs. Among them, Monascus‐fermented products have been used as traditional Chinese medicine for their anti‐cholesterol effects (Heber et al., 1999). Additionally, pu‐erh tea fermented with *Monascus purpureus* displays a better hypolipidemic effect than its non‐fermented raw material (Deng et al., 2021). In our study, MSS showed anti‐obesity effects and improved the lipid profile. These findings may contribute to the treatment of obesity and hyperlipidemia in humans.

To further elucidate the underlying molecular mechanism for the effect of MSS on the regulation of lipid metabolism, we investigated changes in the genes and proteins associated with the AMPK signaling pathway. Since lipogenesis plays a central role in the accumulation of lipids in the liver of HFD‐fed rats (Zhang et al., 2013), this study mainly focused on the enzymes involved in hepatic lipogenesis, which are regulated by the AMPK signaling pathway. The AMPK signaling pathway is known to play a crucial role in the regulation of hepatic lipid metabolism (Yang et al., 2015). AMPK is an important kinase that regulates energy homeostasis and plays a critical role in regulating energy homeostasis. The activation of AMPK is invaluable for the treatment of hyperlipidemia, obesity, and related complications (Steinberg & Carling, 2019). Some reports have indicated that new drugs that activate AMPK may have therapeutic potential against hyperlipidemia, obesity, and related metabolic syndromes (Madhavi et al., 2019). AMPK downregulates the cleavage processing and transcription activity of SREBP while SREBP‐1c binds to the promoter regions of genes such as *ACC, FAS*, and *SCD1* (Currie et al., 2013), which are closely related to fat synthesis. It is well‐known that ACC and FAS are critical enzymes for synthesizing fatty acids, and they serve as markers of the therapeutic effect in hyperlipidemia (Kemp et al., 2003). Among them, ACC catalyzes the synthesis of malonyl‐CoA from acetyl‐CoA, which is the first key step of de novo fatty acid synthesis, and FAS catalyzes the biosynthesis of long‐chain fatty acids. Dietary bioactive compounds such as oxyresveratrol (Tan et al., 2017), tomato extract (Choi et al., 2013), theaflavin TF3 (Zhang et al., 2020), and sulforaphane (Choi et al., 2014) can improve obesity and reduce lipid accumulation by activating the AMPK signaling pathway. In the preventive study, the HFD1 group exhibited a significantly increased gene and protein expression of SREBP‐1c, ACC, and FAS while no significant effect was observed on AMPKα gene and protein expression in HFD1 group than in ND1 group. This is probably due to the duration of administration of HFD, which was not long enough to produce any major changes. After intervention with MSS, the gene and protein expression levels of AMPKα upregulated while the expression of SREBP‐1c, ACC, and FAS decreased. Similarly, in the treatment study, MSS showed a similar ability to activate AMPK expression, which inhibited the expression of SREBP‐1c, ACC, and FAS in rats. Taken together, these results demonstrated that MSS could regulate lipid metabolism by decreasing hepatic lipid synthesis through modulation of the AMPK signaling pathway, thereby preventing and treating obesity and hyperlipidemia. Additionally, the reduction in body weight can be interpreted by the effect of lipolysis (Baselga‐Escudero et al., 2015). Studies have shown that proanthocyanidins enhance lipid degradation by activating β‐oxidation, which reduces lipid accumulation in the liver (Caimari et al., 2013). Moreover, catalpol effectively suppresses lipogenesis and enhances lipolysis through the AMPK signaling pathway, thus, decreasing the liver lipid accumulation (Tian et al., 2020). In our study, we found that MSS treatment reduced the body weight of already obese rats accompanied by a decrease in aortic lipid deposition, suggesting that regulating lipid metabolism and reducing lipid levels could develop through many different pathways in addition to reducing lipid synthesis, such as promoting lipid transport, clearance, and excretion. Thus, the ultimate goal of prevention and treatment of hyperlipidemia can be achieved. In future, we will further examine the underlying mechanism of MSS in preventing and treating hyperlipidemia from the aspects of lipid transport, clearance, and excretion.

## 5 CONCLUDING REMARKS

Overall, our current study demonstrated that MSS could prevent HFD‐induced obesity and hyperlipidemia in rats, and the underlying mechanism may be associated with the AMPK signaling pathway. Furthermore, our results demonstrated the therapeutic potential of MSS on hyperlipidemia in obese rats, and the underlying mechanism may be dependent on activation of the AMPK signaling pathway. Thus, further studies should investigate the potential molecular mechanisms of MSS against obesity and its metabolic complications.

## 6 ETHICS DECLARATIONS

All animal experiments were conducted in accordance with the Affairs Concerning Experimental Animals, and they were approved by the Guizhou Medical University Animal Ethics Committee (Ethics No. 2,000,221).

## CONFLICT OF INTEREST

The authors have no conflicts of interest to declare.

## AUTHOR CONTRIBUTIONS

**Hongmei Yang:** Conceptualization (equal); Data curation (equal); Formal analysis (equal); Investigation (equal); Methodology (equal); Resources (equal); Visualization (lead); Writing‐original draft (lead). **Jiao** **Xie:** Data curation (equal); Visualization (supporting); Writing‐review & editing (equal). **Nanlan Wang:** Supervision (equal); Validation (equal); Writing‐review & editing (equal). **Qianqian Zhou:** Investigation (equal). **Yang Lu:** Conceptualization (equal); Investigation (equal); Methodology (equal); Software (equal). **Zihan Qu:** Conceptualization (equal); Investigation (equal); Methodology (equal). **Huiqun Wang:** Conceptualization (equal); Funding acquisition (equal); Methodology (equal); Project administration (equal); Supervision (equal); Validation (equal); Writing‐review & editing (lead).
